# *cis*-Dichloridobis[diphen­yl(4-vinyl­phenyl)phosphane-κ*P*]platinum(II)

**DOI:** 10.1107/S1600536811043789

**Published:** 2011-11-05

**Authors:** Hezron Ogutu, Reinout Meijboom

**Affiliations:** a Research Centre for Synthesis and Catalysis, Department of Chemistry, University of Johannesburg, PO Box 524, Auckland Park, 2006 Johannesburg, South Africa

## Abstract

The title compound, [PtCl_2_(C_20_H_17_P)_2_], forms a monomeric *cis*-square-planar geometry. The Pt—P bond lengths are 2.2489 (9) and 2.2627 (9) Å, whereas the Pt—Cl bond lengths are 2.3566 (9) and 2.3336 (9) Å.

## Related literature

For a review of related compounds, see: Spessard & Miessler (1996 ▶). For the structure of *trans*-dichloridobis[diphen­yl(4-vinyl­phen­yl)phos­phane]palladium(II), see: Meijboom (2011 ▶). For the synthesis of the starting materials, see: Drew & Doyle (1990 ▶).
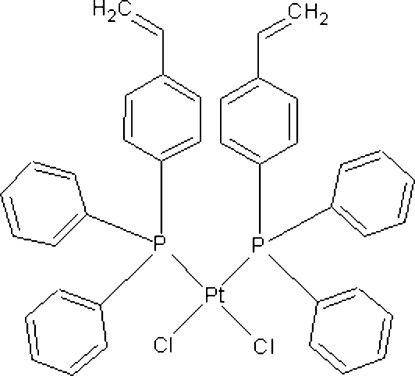


## Experimental

### 

#### Crystal data

[PtCl_2_(C_20_H_17_P)_2_]
*M* *_r_* = 842.6
Triclinic, 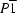

*a* = 10.0670 (5) Å
*b* = 12.7080 (7) Å
*c* = 14.4200 (7) Å
α = 100.179 (3)°
β = 97.519 (3)°
γ = 108.465 (3)°
*V* = 1687.42 (15) Å^3^
*Z* = 2
Cu *K*α radiation
μ = 10.34 mm^−1^
*T* = 173 K
0.09 × 0.05 × 0.05 mm

#### Data collection

Bruker APEXII CCD diffractometer
Absorption correction: multi-scan (*SADABS*; Bruker; 2004 ▶) *T*_min_ = 0.565, *T*_max_ = 0.596
33523 measured reflections
5650 independent reflections
5076 reflections with *I* > 2σ(*I*)
*R*_int_ = 0.066

#### Refinement

*R*[*F* ^2^ > 2σ(*F* ^2^)] = 0.027
*wR*(*F* ^2^) = 0.065
*S* = 1.08
5650 reflections
406 parameters
H-atom parameters constrained
Δρ_max_ = 1.20 e Å^−3^
Δρ_min_ = −0.65 e Å^−3^

### 

Data collection: *APEX2* (Bruker, 2005 ▶); cell refinement: *SAINT* (Bruker, 2004 ▶); data reduction: *SAINT*; program(s) used to solve structure: *SHELXS97* (Sheldrick, 2008 ▶); program(s) used to refine structure: *SHELXL97* (Sheldrick, 2008 ▶); molecular graphics: *SHELXTL* (Sheldrick, 2008 ▶); software used to prepare material for publication: *SHELXTL*.

## Supplementary Material

Crystal structure: contains datablock(s) global, I. DOI: 10.1107/S1600536811043789/kp2359sup1.cif

Structure factors: contains datablock(s) I. DOI: 10.1107/S1600536811043789/kp2359Isup2.hkl

Additional supplementary materials:  crystallographic information; 3D view; checkCIF report

## Figures and Tables

**Fig. 1.**
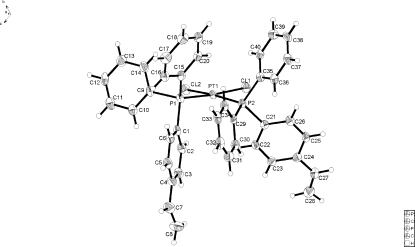
The structure of the title compound, showing 50% probability displacement ellipsoids. For the C atoms, the first digit indicates ring number and the second digit indicates the position of the atom in the ring. Some labels have been omitted for clarity, all rings have been numbered in the same, systematic manner. H atoms are depicted by arbitrary size spheres. Hashed atoms are generated by symmetry (-x, -y, 1 - z).

## supplementary crystallographic information

### Comment

Transition metal complexes containing phosphine, arsine and stibine ligands are widely being investigated in various fields of organometallic chemistry (Spessard & Miessler, 1996). As part of a systematic investigation involving complexes with the general formula *trans/cis*-[*MX*_2_(*L*)_2_] (*M* = Pt or Pd; *X* = halogen, Me, Ph; *L* = group 15 donor ligand), the crystals of the title compound, were obtained.

[PtCl_2_(*L*)_2_] (*L* = tertiary phosphine, arsine or stibine) complexes can conveniently be prepared by the substitution of 1,5-cyclooctadiene (COD) from [PtCl_2_(COD)]. The title compound, *cis*-[PtCl_2_{P(4—H_2_C=CHC_6_H_4_) Ph_2_}_2_], crystallizes in the triclinic spacegroup *P*1, with the Pt atom on a center of symmetry and each pair of equivalent ligands in a *cis* orientation. The geometry is a slightly distorted square planar and the Pt atom is slightly elevated out of the coordinating atom plane. The two P atoms are closer to each other but away from the two chloride atoms with angles of P1—Pt—P2 = 96.1 (4)° and Cl1—Pt—Cl2 = 87.7 (4)° whereas the P1—Pt—Cl1 is = 175.1 (4)° and that of P1—Pt—Cl2 being 89.6 (4)°

The title compound compares well with other closely related Pt^II^ complexes from the literature containing two chloro and two tertiary phosphine ligands in a *cis* geometry. The title compound, containing Pt—Cl bond lengths of 2.3566 (9) and 2.3336 (9) Å and Pt—P bond distances of 2.2489 (9) and 2.2627 (9) Å, fits well into the typical range for complexes of this kind. Notably the title compound did not crystallise as a solvated complex; these type of Pt^II^ complexes have a tendency to crystallise as solvates (Meijboom & Omondi, 2011).

Large thermal vibrations on the periphery of the molecule results in a badly defined C═C bond length. Disordered modelling resulted in an unstable refinement.

### Experimental

Diphenylphosphinostyrene (0.05 g, 0.35 mmol) was dissolved in acetone (5 ml). A solution of [Pt(COD)Cl_2_] (0.05 g, 0.17 mmol) in acetone (5 ml) was added to the phosphine solution. The mixture was stirred for 5 min, after which the solution was left to crystallise. Yellow crystals of the title compound were obtained.

### Refinement

The aromatic H atoms were placed in geometrically idealized positions (C—H = 0.95–0.98) and constrained to ride on their parent atoms with *U*_iso_(H) = 1.2*U*_eq_(C).

### Crystal data

**Table:**

[PtCl_2_(C_20_H_17_P)_2_]	*Z* = 2
*M**_r_* = 842.6	*F*(000) = 832
Triclinic, *P*1	*D*_x_ = 1.658 Mg m^−^^3^
Hall symbol: -P 1	Cu *K*α radiation, λ = 1.54184 Å
*a* = 10.0670 (5) Å	Cell parameters from 9915 reflections
*b* = 12.7080 (7) Å	θ = 3.2–65.5°
*c* = 14.4200 (7) Å	µ = 10.34 mm^−^^1^
α = 100.179 (3)°	*T* = 173 K
β = 97.519 (3)°	Rectagular, colourless
γ = 108.465 (3)°	0.09 × 0.05 × 0.05 mm
*V* = 1687.42 (15) Å^3^	

### Data collection

**Table:**

Bruker APEXII CCD diffractometer	5076 reflections with *I* > 2σ(*I*)
graphite	*R*_int_ = 0.066
φ and ω scans	θ_max_ = 66.0°, θ_min_ = 3.2°
Absorption correction: multi-scan (*SADABS*; Bruker; 2004)	*h* = −11→9
*T*_min_ = 0.565, *T*_max_ = 0.596	*k* = −14→15
33523 measured reflections	*l* = −16→16
5650 independent reflections	

### Refinement

**Table:**

Refinement on *F*^2^	Primary atom site location: structure-invariant direct methods
Least-squares matrix: full	Secondary atom site location: difference Fourier map
*R*[*F*^2^ > 2σ(*F*^2^)] = 0.027	Hydrogen site location: inferred from neighbouring sites
*wR*(*F*^2^) = 0.065	H-atom parameters constrained
*S* = 1.08	*w* = 1/[σ^2^(*F*_o_^2^) + (0.0347*P*)^2^] where *P* = (*F*_o_^2^ + 2*F*_c_^2^)/3
5650 reflections	(Δ/σ)_max_ = 0.001
406 parameters	Δρ_max_ = 1.20 e Å^−^^3^
0 restraints	Δρ_min_ = −0.65 e Å^−^^3^

### Special details

**Table:**

Geometry. All e.s.d.'s (except the e.s.d. in the dihedral angle between two l.s. planes) are estimated using the full covariance matrix. The cell e.s.d.'s are taken into account individually in the estimation of e.s.d.'s in distances, angles and torsion angles; correlations between e.s.d.'s in cell parameters are only used when they are defined by crystal symmetry. An approximate (isotropic) treatment of cell e.s.d.'s is used for estimating e.s.d.'s involving l.s. planes.
Refinement. Refinement of *F*^2^ against ALL reflections. The weighted *R*-factor *wR* and goodness of fit *S* are based on *F*^2^, conventional *R*-factors *R* are based on *F*, with *F* set to zero for negative *F*^2^. The threshold expression of *F*^2^ > σ(*F*^2^) is used only for calculating *R*-factors(gt) *etc*. and is not relevant to the choice of reflections for refinement. *R*-factors based on *F*^2^ are statistically about twice as large as those based on *F*, and *R*-factors based on ALL data will be even larger.

### Fractional atomic coordinates and isotropic or equivalent isotropic displacement parameters (Å^2^)

**Table:**

	*x*	*y*	*z*	*U*_iso_*/*U*_eq_	
Pt1	0.479071 (15)	0.400360 (13)	0.732175 (11)	0.01954 (7)	
Cl2	0.42103 (10)	0.54124 (8)	0.82770 (7)	0.0267 (2)	
P1	0.70052 (10)	0.46884 (8)	0.82343 (7)	0.0208 (2)	
Cl1	0.25050 (9)	0.34413 (8)	0.63416 (7)	0.0264 (2)	
P2	0.50511 (10)	0.24668 (8)	0.64094 (7)	0.0206 (2)	
C20	0.7955 (4)	0.5146 (3)	0.6588 (3)	0.0260 (9)	
H20	0.6989	0.4949	0.633	0.031*	
C19	0.8965 (4)	0.5507 (3)	0.6033 (3)	0.0299 (10)	
H19	0.8677	0.558	0.5415	0.036*	
C8	0.6325 (5)	0.0769 (4)	1.1108 (4)	0.0475 (13)	
H8A	0.5405	0.0761	1.0908	0.057*	
H8B	0.6483	0.0306	1.151	0.057*	
C26	0.2685 (4)	0.0511 (3)	0.5411 (3)	0.0249 (9)	
H26	0.3027	0.0614	0.4853	0.03*	
C38	0.5641 (4)	0.2698 (4)	0.3332 (3)	0.0308 (10)	
H38	0.5772	0.2752	0.2714	0.037*	
C5	0.8494 (4)	0.2706 (3)	0.9791 (3)	0.0290 (9)	
H5	0.932	0.2536	0.9934	0.035*	
C1	0.7243 (4)	0.3699 (3)	0.8967 (3)	0.0232 (9)	
C3	0.6105 (4)	0.2494 (3)	0.9965 (3)	0.0285 (9)	
H3	0.5316	0.219	1.0231	0.034*	
C2	0.6046 (4)	0.3212 (3)	0.9359 (3)	0.0270 (9)	
H2	0.5216	0.3375	0.9209	0.032*	
C31	0.7325 (5)	0.0723 (4)	0.7643 (3)	0.0349 (10)	
H31	0.7139	0.0134	0.7963	0.042*	
C29	0.6489 (4)	0.1963 (3)	0.6840 (3)	0.0213 (8)	
C9	0.7452 (4)	0.6041 (3)	0.9114 (3)	0.0232 (9)	
C16	0.9833 (4)	0.5385 (3)	0.7908 (3)	0.0300 (10)	
H16	1.0135	0.5378	0.8544	0.036*	
C21	0.3435 (4)	0.1247 (3)	0.6294 (3)	0.0212 (8)	
C4	0.7321 (4)	0.2213 (4)	1.0186 (3)	0.0292 (9)	
C18	1.0386 (5)	0.5756 (4)	0.6399 (3)	0.0357 (11)	
H18	1.1058	0.5962	0.6017	0.043*	
C11	0.7848 (4)	0.7147 (4)	1.0721 (3)	0.0321 (10)	
H11	0.7909	0.7191	1.1376	0.039*	
C35	0.5259 (4)	0.2551 (3)	0.5190 (3)	0.0232 (8)	
C40	0.4941 (4)	0.3385 (3)	0.4785 (3)	0.0241 (9)	
H40	0.4598	0.3895	0.5135	0.029*	
C6	0.8463 (4)	0.3438 (3)	0.9193 (3)	0.0264 (9)	
H6	0.9263	0.3756	0.8943	0.032*	
C25	0.1439 (4)	−0.0369 (3)	0.5366 (3)	0.0255 (9)	
H25	0.0948	−0.085	0.4772	0.031*	
C17	1.0824 (4)	0.5701 (4)	0.7338 (3)	0.0356 (11)	
H17	1.179	0.5877	0.7584	0.043*	
C15	0.8377 (4)	0.5078 (3)	0.7529 (3)	0.0244 (9)	
C24	0.0888 (4)	−0.0562 (3)	0.6182 (3)	0.0258 (9)	
C34	0.7866 (4)	0.2447 (3)	0.6668 (3)	0.0252 (9)	
H34	0.8051	0.302	0.6331	0.03*	
C23	0.1657 (4)	0.0167 (3)	0.7063 (3)	0.0246 (9)	
H23	0.1329	0.0048	0.7623	0.029*	
C33	0.8950 (4)	0.2073 (4)	0.6999 (3)	0.0299 (10)	
H33	0.9866	0.2405	0.6893	0.036*	
C13	0.7992 (5)	0.8069 (4)	0.9414 (3)	0.0368 (11)	
H13	0.8147	0.873	0.9187	0.044*	
C39	0.5136 (4)	0.3451 (4)	0.3863 (3)	0.0302 (10)	
H39	0.4926	0.4009	0.3597	0.036*	
C30	0.6241 (4)	0.1105 (3)	0.7333 (3)	0.0261 (9)	
H30	0.5335	0.078	0.7457	0.031*	
C32	0.8679 (4)	0.1212 (4)	0.7482 (3)	0.0317 (10)	
H32	0.9408	0.096	0.77	0.038*	
C7	0.7408 (5)	0.1439 (4)	1.0819 (3)	0.0397 (11)	
H7	0.8309	0.1419	1.1037	0.048*	
C14	0.7678 (4)	0.7031 (4)	0.8781 (3)	0.0315 (10)	
H14	0.7618	0.6996	0.8127	0.038*	
C37	0.5949 (4)	0.1867 (4)	0.3722 (3)	0.0316 (10)	
H37	0.628	0.1355	0.3364	0.038*	
C22	0.2897 (4)	0.1063 (3)	0.7116 (3)	0.0232 (8)	
H22	0.3381	0.155	0.771	0.028*	
C10	0.7534 (4)	0.6105 (4)	1.0099 (3)	0.0277 (9)	
H10	0.7377	0.5448	1.0333	0.033*	
C28	−0.1179 (5)	−0.1685 (4)	0.6776 (4)	0.0447 (12)	
H28A	−0.0816	−0.1236	0.7397	0.054*	
H28B	−0.2045	−0.2289	0.664	0.054*	
C12	0.8073 (4)	0.8125 (4)	1.0382 (3)	0.0343 (10)	
H12	0.8281	0.8823	1.0809	0.041*	
C36	0.5768 (4)	0.1795 (3)	0.4645 (3)	0.0273 (9)	
H36	0.5987	0.1238	0.4907	0.033*	
C27	−0.0474 (4)	−0.1469 (4)	0.6092 (3)	0.0343 (10)	
H27	−0.0884	−0.1944	0.5483	0.041*	

### Atomic displacement parameters (Å^2^)

**Table:**

	*U*^11^	*U*^22^	*U*^33^	*U*^12^	*U*^13^	*U*^23^
Pt1	0.01963 (9)	0.02256 (10)	0.01699 (10)	0.00948 (7)	0.00360 (7)	0.00210 (7)
Cl2	0.0297 (5)	0.0311 (5)	0.0222 (5)	0.0169 (4)	0.0065 (4)	0.0010 (4)
P1	0.0201 (4)	0.0247 (5)	0.0165 (5)	0.0091 (4)	0.0020 (4)	0.0015 (4)
Cl1	0.0208 (4)	0.0294 (5)	0.0276 (5)	0.0102 (4)	0.0007 (4)	0.0031 (4)
P2	0.0225 (5)	0.0211 (5)	0.0181 (5)	0.0093 (4)	0.0037 (4)	0.0011 (4)
C20	0.0250 (19)	0.023 (2)	0.027 (2)	0.0070 (17)	0.0048 (18)	0.0009 (18)
C19	0.036 (2)	0.026 (2)	0.025 (2)	0.0087 (19)	0.0090 (19)	0.0021 (19)
C8	0.050 (3)	0.052 (3)	0.050 (3)	0.021 (2)	0.010 (3)	0.029 (3)
C26	0.027 (2)	0.028 (2)	0.022 (2)	0.0146 (18)	0.0067 (18)	0.0024 (18)
C38	0.034 (2)	0.039 (3)	0.016 (2)	0.008 (2)	0.0080 (18)	0.006 (2)
C5	0.029 (2)	0.033 (2)	0.025 (2)	0.0157 (19)	−0.0003 (18)	0.003 (2)
C1	0.026 (2)	0.024 (2)	0.016 (2)	0.0093 (17)	0.0018 (16)	−0.0026 (17)
C3	0.032 (2)	0.032 (2)	0.021 (2)	0.0113 (19)	0.0079 (18)	0.0022 (19)
C2	0.025 (2)	0.030 (2)	0.024 (2)	0.0123 (18)	0.0006 (17)	0.0016 (19)
C31	0.040 (2)	0.027 (2)	0.036 (3)	0.014 (2)	−0.001 (2)	0.006 (2)
C29	0.0272 (19)	0.018 (2)	0.016 (2)	0.0106 (16)	0.0004 (16)	−0.0040 (17)
C9	0.0195 (18)	0.029 (2)	0.018 (2)	0.0095 (16)	−0.0017 (16)	−0.0015 (18)
C16	0.027 (2)	0.032 (2)	0.028 (2)	0.0097 (18)	0.0002 (18)	0.003 (2)
C21	0.0203 (18)	0.023 (2)	0.022 (2)	0.0113 (16)	0.0028 (17)	0.0042 (18)
C4	0.036 (2)	0.029 (2)	0.022 (2)	0.0126 (19)	0.0046 (19)	0.0049 (19)
C18	0.033 (2)	0.034 (3)	0.040 (3)	0.009 (2)	0.019 (2)	0.005 (2)
C11	0.027 (2)	0.043 (3)	0.024 (2)	0.0144 (19)	0.0074 (18)	−0.002 (2)
C35	0.0212 (18)	0.026 (2)	0.021 (2)	0.0077 (16)	0.0035 (16)	0.0033 (18)
C40	0.0248 (19)	0.022 (2)	0.023 (2)	0.0076 (17)	0.0057 (17)	−0.0002 (18)
C6	0.025 (2)	0.029 (2)	0.022 (2)	0.0092 (17)	0.0030 (17)	−0.0005 (19)
C25	0.030 (2)	0.024 (2)	0.020 (2)	0.0118 (18)	0.0016 (17)	−0.0018 (18)
C17	0.024 (2)	0.038 (3)	0.046 (3)	0.0110 (19)	0.010 (2)	0.009 (2)
C15	0.027 (2)	0.023 (2)	0.023 (2)	0.0104 (17)	0.0060 (17)	0.0011 (18)
C24	0.030 (2)	0.021 (2)	0.028 (2)	0.0126 (17)	0.0046 (18)	0.0049 (19)
C34	0.029 (2)	0.026 (2)	0.020 (2)	0.0124 (17)	0.0042 (17)	−0.0017 (18)
C23	0.027 (2)	0.026 (2)	0.022 (2)	0.0110 (17)	0.0058 (17)	0.0049 (18)
C33	0.0217 (19)	0.037 (3)	0.025 (2)	0.0105 (18)	0.0030 (18)	−0.008 (2)
C13	0.044 (3)	0.028 (2)	0.037 (3)	0.015 (2)	0.004 (2)	0.002 (2)
C39	0.032 (2)	0.030 (2)	0.031 (2)	0.0119 (19)	0.0059 (19)	0.009 (2)
C30	0.025 (2)	0.026 (2)	0.025 (2)	0.0103 (17)	0.0037 (18)	0.0012 (19)
C32	0.029 (2)	0.032 (2)	0.033 (2)	0.0168 (19)	−0.0010 (19)	−0.002 (2)
C7	0.039 (2)	0.044 (3)	0.038 (3)	0.017 (2)	0.003 (2)	0.013 (2)
C14	0.037 (2)	0.032 (2)	0.023 (2)	0.0128 (19)	0.0046 (19)	0.002 (2)
C37	0.033 (2)	0.036 (3)	0.024 (2)	0.0118 (19)	0.0082 (19)	0.001 (2)
C22	0.029 (2)	0.023 (2)	0.017 (2)	0.0108 (17)	0.0021 (17)	0.0009 (17)
C10	0.0238 (19)	0.032 (2)	0.025 (2)	0.0108 (18)	0.0047 (18)	0.0007 (19)
C28	0.043 (3)	0.037 (3)	0.043 (3)	−0.001 (2)	0.009 (2)	0.008 (2)
C12	0.033 (2)	0.033 (3)	0.029 (3)	0.0117 (19)	0.003 (2)	−0.010 (2)
C36	0.027 (2)	0.029 (2)	0.025 (2)	0.0104 (18)	0.0046 (18)	0.0011 (19)
C27	0.035 (2)	0.029 (2)	0.029 (2)	0.0055 (19)	−0.001 (2)	0.001 (2)

### Geometric parameters (Å, °)

**Table:**

Pt1—P1	2.2489 (9)	C16—H16	0.93
Pt1—P2	2.2627 (9)	C21—C22	1.392 (5)
Pt1—Cl2	2.3336 (9)	C4—C7	1.470 (6)
Pt1—Cl1	2.3566 (9)	C18—C17	1.389 (6)
P1—C15	1.817 (4)	C18—H18	0.93
P1—C1	1.829 (4)	C11—C12	1.378 (6)
P1—C9	1.832 (4)	C11—C10	1.378 (6)
P2—C35	1.815 (4)	C11—H11	0.93
P2—C21	1.822 (4)	C35—C40	1.397 (6)
P2—C29	1.840 (4)	C35—C36	1.398 (5)
C20—C19	1.390 (5)	C40—C39	1.383 (5)
C20—C15	1.394 (6)	C40—H40	0.93
C20—H20	0.93	C6—H6	0.93
C19—C18	1.372 (6)	C25—C24	1.392 (5)
C19—H19	0.93	C25—H25	0.93
C8—C7	1.320 (6)	C17—H17	0.93
C8—H8A	0.93	C24—C23	1.394 (6)
C8—H8B	0.93	C24—C27	1.457 (6)
C26—C25	1.376 (5)	C34—C33	1.385 (6)
C26—C21	1.396 (5)	C34—H34	0.93
C26—H26	0.93	C23—C22	1.380 (5)
C38—C37	1.378 (6)	C23—H23	0.93
C38—C39	1.382 (6)	C33—C32	1.373 (6)
C38—H38	0.93	C33—H33	0.93
C5—C6	1.380 (6)	C13—C12	1.375 (6)
C5—C4	1.394 (6)	C13—C14	1.382 (6)
C5—H5	0.93	C13—H13	0.93
C1—C6	1.385 (5)	C39—H39	0.93
C1—C2	1.409 (5)	C30—H30	0.93
C3—C2	1.380 (6)	C32—H32	0.93
C3—C4	1.394 (6)	C7—H7	0.93
C3—H3	0.93	C14—H14	0.93
C2—H2	0.93	C37—C36	1.381 (6)
C31—C32	1.376 (6)	C37—H37	0.93
C31—C30	1.382 (6)	C22—H22	0.93
C31—H31	0.93	C10—H10	0.93
C29—C30	1.380 (5)	C28—C27	1.307 (6)
C29—C34	1.402 (5)	C28—H28A	0.93
C9—C14	1.388 (6)	C28—H28B	0.93
C9—C10	1.399 (5)	C12—H12	0.93
C16—C17	1.382 (6)	C36—H36	0.93
C16—C15	1.397 (5)	C27—H27	0.93
			
P1—Pt1—P2	96.14 (3)	C40—C35—C36	118.7 (4)
P1—Pt1—Cl2	89.61 (3)	C40—C35—P2	120.7 (3)
P2—Pt1—Cl2	171.93 (3)	C36—C35—P2	120.5 (3)
P1—Pt1—Cl1	175.16 (3)	C39—C40—C35	119.9 (4)
P2—Pt1—Cl1	86.92 (3)	C39—C40—H40	120.1
Cl2—Pt1—Cl1	87.72 (3)	C35—C40—H40	120.1
C15—P1—C1	113.60 (18)	C5—C6—C1	120.1 (4)
C15—P1—C9	100.60 (18)	C5—C6—H6	119.9
C1—P1—C9	103.91 (18)	C1—C6—H6	119.9
C15—P1—Pt1	112.17 (13)	C26—C25—C24	122.1 (4)
C1—P1—Pt1	110.37 (13)	C26—C25—H25	118.9
C9—P1—Pt1	115.71 (12)	C24—C25—H25	118.9
C35—P2—C21	105.89 (17)	C16—C17—C18	120.4 (4)
C35—P2—C29	103.14 (17)	C16—C17—H17	119.8
C21—P2—C29	103.27 (17)	C18—C17—H17	119.8
C35—P2—Pt1	115.90 (13)	C20—C15—C16	118.9 (4)
C21—P2—Pt1	107.66 (12)	C20—C15—P1	118.0 (3)
C29—P2—Pt1	119.57 (12)	C16—C15—P1	122.9 (3)
C19—C20—C15	120.6 (4)	C25—C24—C23	117.5 (4)
C19—C20—H20	119.7	C25—C24—C27	120.0 (4)
C15—C20—H20	119.7	C23—C24—C27	122.4 (4)
C18—C19—C20	119.8 (4)	C33—C34—C29	120.1 (4)
C18—C19—H19	120.1	C33—C34—H34	119.9
C20—C19—H19	120.1	C29—C34—H34	119.9
C7—C8—H8A	120	C22—C23—C24	121.0 (4)
C7—C8—H8B	120	C22—C23—H23	119.5
H8A—C8—H8B	120	C24—C23—H23	119.5
C25—C26—C21	119.9 (4)	C32—C33—C34	120.3 (4)
C25—C26—H26	120.1	C32—C33—H33	119.8
C21—C26—H26	120.1	C34—C33—H33	119.8
C37—C38—C39	119.9 (4)	C12—C13—C14	119.9 (4)
C37—C38—H38	120.1	C12—C13—H13	120
C39—C38—H38	120.1	C14—C13—H13	120
C6—C5—C4	121.8 (4)	C38—C39—C40	120.8 (4)
C6—C5—H5	119.1	C38—C39—H39	119.6
C4—C5—H5	119.1	C40—C39—H39	119.6
C6—C1—C2	119.0 (4)	C29—C30—C31	120.7 (4)
C6—C1—P1	127.0 (3)	C29—C30—H30	119.7
C2—C1—P1	113.9 (3)	C31—C30—H30	119.7
C2—C3—C4	121.4 (4)	C33—C32—C31	119.9 (4)
C2—C3—H3	119.3	C33—C32—H32	120.1
C4—C3—H3	119.3	C31—C32—H32	120.1
C3—C2—C1	120.0 (4)	C8—C7—C4	126.0 (4)
C3—C2—H2	120	C8—C7—H7	117
C1—C2—H2	120	C4—C7—H7	117
C32—C31—C30	120.3 (4)	C13—C14—C9	120.5 (4)
C32—C31—H31	119.8	C13—C14—H14	119.7
C30—C31—H31	119.8	C9—C14—H14	119.7
C30—C29—C34	118.7 (4)	C38—C37—C36	120.0 (4)
C30—C29—P2	121.1 (3)	C38—C37—H37	120
C34—C29—P2	120.2 (3)	C36—C37—H37	120
C14—C9—C10	119.2 (4)	C23—C22—C21	120.9 (4)
C14—C9—P1	118.4 (3)	C23—C22—H22	119.5
C10—C9—P1	122.4 (3)	C21—C22—H22	119.5
C17—C16—C15	120.0 (4)	C11—C10—C9	119.6 (4)
C17—C16—H16	120	C11—C10—H10	120.2
C15—C16—H16	120	C9—C10—H10	120.2
C22—C21—C26	118.6 (3)	C27—C28—H28A	120
C22—C21—P2	118.7 (3)	C27—C28—H28B	120
C26—C21—P2	122.7 (3)	H28A—C28—H28B	120
C5—C4—C3	117.7 (4)	C13—C12—C11	120.1 (4)
C5—C4—C7	119.8 (4)	C13—C12—H12	120
C3—C4—C7	122.6 (4)	C11—C12—H12	120
C19—C18—C17	120.2 (4)	C37—C36—C35	120.8 (4)
C19—C18—H18	119.9	C37—C36—H36	119.6
C17—C18—H18	119.9	C35—C36—H36	119.6
C12—C11—C10	120.8 (4)	C28—C27—C24	126.9 (4)
C12—C11—H11	119.6	C28—C27—H27	116.6
C10—C11—H11	119.6	C24—C27—H27	116.6
			
P2—Pt1—P1—C15	62.46 (14)	Pt1—P2—C35—C36	164.4 (3)
Cl2—Pt1—P1—C15	−123.21 (14)	C36—C35—C40—C39	−0.2 (6)
P2—Pt1—P1—C1	−65.31 (13)	P2—C35—C40—C39	178.6 (3)
Cl2—Pt1—P1—C1	109.02 (13)	C4—C5—C6—C1	−0.3 (6)
P2—Pt1—P1—C9	177.08 (15)	C2—C1—C6—C5	0.3 (6)
Cl2—Pt1—P1—C9	−8.59 (15)	P1—C1—C6—C5	177.3 (3)
P1—Pt1—P2—C35	−105.47 (14)	C21—C26—C25—C24	−0.3 (6)
Cl1—Pt1—P2—C35	70.77 (14)	C15—C16—C17—C18	2.5 (6)
P1—Pt1—P2—C21	136.24 (13)	C19—C18—C17—C16	0.6 (7)
Cl1—Pt1—P2—C21	−47.53 (13)	C19—C20—C15—C16	0.4 (6)
P1—Pt1—P2—C29	19.01 (15)	C19—C20—C15—P1	176.1 (3)
Cl1—Pt1—P2—C29	−164.76 (15)	C17—C16—C15—C20	−3.0 (6)
C15—C20—C19—C18	2.6 (6)	C17—C16—C15—P1	−178.3 (3)
C15—P1—C1—C6	15.3 (4)	C1—P1—C15—C20	139.0 (3)
C9—P1—C1—C6	−93.1 (4)	C9—P1—C15—C20	−110.6 (3)
Pt1—P1—C1—C6	142.2 (3)	Pt1—P1—C15—C20	13.0 (3)
C15—P1—C1—C2	−167.6 (3)	C1—P1—C15—C16	−45.6 (4)
C9—P1—C1—C2	84.0 (3)	C9—P1—C15—C16	64.8 (4)
Pt1—P1—C1—C2	−40.7 (3)	Pt1—P1—C15—C16	−171.6 (3)
C4—C3—C2—C1	−1.5 (6)	C26—C25—C24—C23	−0.7 (6)
C6—C1—C2—C3	0.5 (6)	C26—C25—C24—C27	176.9 (4)
P1—C1—C2—C3	−176.8 (3)	C30—C29—C34—C33	−0.8 (6)
C35—P2—C29—C30	−134.8 (3)	P2—C29—C34—C33	179.5 (3)
C21—P2—C29—C30	−24.7 (3)	C25—C24—C23—C22	1.7 (6)
Pt1—P2—C29—C30	94.8 (3)	C27—C24—C23—C22	−175.9 (4)
C35—P2—C29—C34	44.9 (3)	C29—C34—C33—C32	1.2 (6)
C21—P2—C29—C34	155.0 (3)	C37—C38—C39—C40	0.2 (6)
Pt1—P2—C29—C34	−85.5 (3)	C35—C40—C39—C38	0.2 (6)
C15—P1—C9—C14	48.5 (3)	C34—C29—C30—C31	−0.5 (6)
C1—P1—C9—C14	166.3 (3)	P2—C29—C30—C31	179.2 (3)
Pt1—P1—C9—C14	−72.6 (3)	C32—C31—C30—C29	1.3 (6)
C15—P1—C9—C10	−133.3 (3)	C34—C33—C32—C31	−0.3 (6)
C1—P1—C9—C10	−15.5 (4)	C30—C31—C32—C33	−0.9 (6)
Pt1—P1—C9—C10	105.7 (3)	C5—C4—C7—C8	−167.7 (5)
C25—C26—C21—C22	0.4 (6)	C3—C4—C7—C8	13.0 (7)
C25—C26—C21—P2	−177.3 (3)	C12—C13—C14—C9	−0.4 (6)
C35—P2—C21—C22	−171.5 (3)	C10—C9—C14—C13	0.5 (6)
C29—P2—C21—C22	80.5 (3)	P1—C9—C14—C13	178.8 (3)
Pt1—P2—C21—C22	−46.9 (3)	C39—C38—C37—C36	−0.6 (6)
C35—P2—C21—C26	6.2 (4)	C24—C23—C22—C21	−1.6 (6)
C29—P2—C21—C26	−101.8 (3)	C26—C21—C22—C23	0.6 (5)
Pt1—P2—C21—C26	130.8 (3)	P2—C21—C22—C23	178.4 (3)
C6—C5—C4—C3	−0.6 (6)	C12—C11—C10—C9	0.4 (6)
C6—C5—C4—C7	−179.9 (4)	C14—C9—C10—C11	−0.5 (6)
C2—C3—C4—C5	1.5 (6)	P1—C9—C10—C11	−178.8 (3)
C2—C3—C4—C7	−179.2 (4)	C14—C13—C12—C11	0.2 (6)
C20—C19—C18—C17	−3.1 (6)	C10—C11—C12—C13	−0.3 (6)
C21—P2—C35—C40	104.9 (3)	C38—C37—C36—C35	0.6 (6)
C29—P2—C35—C40	−147.0 (3)	C40—C35—C36—C37	−0.2 (6)
Pt1—P2—C35—C40	−14.4 (4)	P2—C35—C36—C37	−179.0 (3)
C21—P2—C35—C36	−76.4 (3)	C25—C24—C27—C28	−173.6 (5)
C29—P2—C35—C36	31.8 (4)	C23—C24—C27—C28	3.9 (7)
